# Edible Flowers, Old Tradition or New Gastronomic Trend: A First Look at Consumption in Portugal versus Costa Rica

**DOI:** 10.3390/foods9080977

**Published:** 2020-07-23

**Authors:** Raquel P. F. Guiné, Sofia G. Florença, Keylor Villalobos Moya, Ofélia Anjos

**Affiliations:** 1 CERNAS Research Centre, Polytechnic Institute of Viseu, 3504-510 Viseu, Portugal; raquelguine@esav.ipv.pt; 2 Faculty of Food and Nutrition Sciences, University of Porto, 4200-465 Portugal; sofiaguine@gmail.com; 3 School of Agrarian Sciences, National University of Costa Rica, Heredia, Costa Rica; keylorvm87@gmail.com; 4 Polytechnic Institute of Castelo Branco, 6001-909 Castelo Branco, Portugal; 5 Forest Research Centre, School of Agriculture, University of Lisbon, 1349-017 Lisbon, Portugal

**Keywords:** edible flowers, food security, gourmet kitchen, knowledge, questionnaire survey

## Abstract

This study investigated the knowledge and use of edible flowers (EF) in two countries, Portugal, in Europe, and Costa Rica, in Latin America, and aimed to evaluate the similarities and/or differences regarding the utilization of EF in gastronomy. This work consisted of a questionnaire survey, undertaken on a sample of 290 participants. The results indicate that most people surveyed (87%) have heard about EF but believe there is not enough information about them (96%). Only one third of participants consider there are risks associated with the consumption of EF, being those related to toxicity and pesticides. Significant differences (*p* < 0.05) were found between participants from the two countries but not with different professional areas. About half (48%) of the participants had already consumed EF, mostly for decoration or confection of dishes (77% positive answers) and in salads (75%). The flowers consumed most frequently were chamomile and rose, respectively, in Costa Rica and Portugal. Reasons pointed out to consume EF include decoration, taste, novelty and aroma, while aspects such as nutritional value or antioxidant capacity are prized by fewer consumers. EF were mostly acquired in supermarkets, cultivated at home or collected in the wild. In general, most participants (85%) consider the use of EF in gastronomy interesting, but less than one third (27%) believe we should eat EF more often. Finally, discriminant function analysis revealed that country was the variable for which the differences in the consumption of EF was more pronounced, while education level and age group showed the lowest variability between groups.

## 1. Introduction

The consumption of flowers in ancient time is known, on one hand, for being a part of traditional culinary practices, while being also used in the field of alternative medicines. In civilizations in ancient Rome and Greece, as well as in China, the tradition of using edible flowers (EF) is linked to a historic concept, being their use in food preparation, as aroma enhancers, to add flavor and aesthetic value. For example, roses (*Rosa* spp. L.) were used in ancient Rome to provide flavor and sweetness to dishes, in drinks, salads, purees, omelets, and desserts. In France, during the Middle Ages, flowers of calendula (*Calendula officinalis*) were consumed in a wide variety of salads. Documented use of violets (*Viola odorata* L.) in the 17th century related to their ability to confer sweetness and color to syrups [1,2,3]. 

Although EF have been consumed over the ages, their use is not as widespread as other foods and their inclusion in gastronomic preparations is more often linked to special occasions, gourmet cuisine and certain chefs’ recipes or suggestions. The health and aesthetic properties of EF represent a niche market nowadays; however, from a strategic point of view, the food industry and food service operators might greatly benefit from a wider inclusion of EF in food products, pre-prepared meals, drinks, freshly prepared dishes and desserts, as a way of positive differentiation and service enhancement [4,5]. 

According to a review by Fernandes et al. [6] about the benefits of EF for human health, they possess nutritional value—being rich in moisture, carbohydrates and protein, and being low in lipids. They also contain interesting amounts of ash, including dietary minerals such as calcium, iron, potassium, magnesium, phosphorous or zinc. Furthermore, they contain bioactive components, such as phenolic compounds, which contribute to their high antioxidant activity, while also conferring color and aroma. Other biological effects include antimicrobial and anti-inflammatory activities which are also reported to inhibit cell proliferation, turning them into a potential ally for cancer treatment and prevention [7,8,9,10]. Still, it is important to bear in mind what amounts need to be ingested for these health effects to be effective on the human body. From this point of view, many of these possible health claims are not yet established through recommended intake dosages.

As with other food categories, the consumption of edible flowers involves a complex decision process, when making food choices. These food choices are related to the products’ characteristics but also to the individual’s history and context, including integration of personal ideas, resources and social influences with the social, cultural and physical environments [11,12,13,14,15,16]. With regard to EF, some people consider the unique combination of their pleasant visual aspect, color, aroma, taste, shape and nutrition, consequently invoking these reasons when deciding to consume EF. On the other hand, some people value their nutritional composition more and them being fresh, unprocessed or minimally processed food products, making these the reasons for eating them [4,17].

This study was carried out to assess the knowledge and use of EF in two countries situated in different parts of the globe, Portugal in Europe and Costa Rica in Latin America, possibly representing different realities regarding the utilization of EF in gastronomy. 

## 2. Materials and Methods

### 2.1. Data Collection and Sample Characterization

This survey was based on a questionnaire that was applied to a convenience sample in two countries, Portugal and Costa Rica. The convenience sample was chosen according to the facility to recruit and place of residence, and it was intended to have a minimum of approximately 150 responses in each of the countries. Therefore, the questionnaire was sent to a high number of people in each of the countries, but the responses obtained were limited in number, a little higher in Portugal than in Costa Rica (151 and 139, respectively). Convenience samples have both the advantages of easy recruitment and not allowing generalization according to estimates of sociodemographic differences. In addition, they can be a good tool for exploratory research [18,19,20,21]. All data collected were treated with confidentiality and met all ethical issues, so that it was impossible to link the answers to a particular individual. The survey was conducted with adult participants only, who answered the questionnaire voluntarily. The data collection was approved by the Ethical Committee at Polytechnic Institute of Viseu and all participants gave explicit consent prior to the data collection.

This survey included 290 participants, of which 47.9% were from Costa Rica and 52.1% were from Portugal, leading to an even distribution between countries (Table 1). The average age of the participants was 40 ± 13 years, slightly higher in Portugal (41 ± 13 years) than in Costa Rica (38 ± 12 years). Most of the participants were aged between 31 and 50 years (52.7%), and the elderly were those least represented (2.1%). Regarding the highest level of education, most of the participants had completed a university degree (83.1%), however, fewer had just completed basic education (3.1%). As for the living environment, the great majority lived in urban (67.2%), followed by rural (22.8%) and then suburban (10.0%) environment. For the combined results of both countries, for all sociodemographic variables considered, a relative homogeneity between the samples from Portugal and Costa Rica was observed—the sex variable was an exception, for which the percentage of men was considerably higher in Costa Rica (37.4%) than Portugal (19.2%).

The professional area of the participants (either regarding work or studies) was also assessed considering its possible influence on the level of information and consumptions habits towards EF. The results in Table 2 indicate that, while in Portugal a high percentage of participants had professional areas related with food and nutrition (58.0%), in Costa Rica the percentage for these areas was much smaller (only 17.3%). Additionally, the professionals linked with restaurants and hotels were more frequent in Portugal (12.1%) than in Costa Rica (5.0%). Regarding professions or studies in the domain of agriculture or agricultural sciences, both countries had a similar representation (31.2% in Costa Rica and 29.3% in Portugal).

### 2.2. Data Analysis

Exploratory analysis was done using SPSS software V25 (IBM, United States Inc.). The crosstabs and chi-square test were used to evaluate possible relations between some of the categorical variables studied at a level of significance of 5%. The coefficient Cramer’s V was used to express the strength of the significant relations found between variables. This coefficient varies, ranging from 0 (no association) to 1 (perfect association), and for V ≈ 0.1 the association is considered weak, V ≈ 0.3 the association is moderate and V ≈ 0.5 or higher, the association is strong [22]. When the conditions did not allow the use of the chi-square test, the Fisher’s exact test was used.

Discriminant function analysis (DFA) was performed using statistic software from StatSoft (Vs 7.09). This is a statistical procedure that classifies unknown individuals and estimates the probability of their classification into a certain group (for example sex or country). DFA assumes a normal distribution for the sample, and the subsequent probability and typicality probability are applied to calculate the classification probabilities [23].

## 3. Results and Discussion

### 3.1. Information about Edible Flowers

Flowers have traditionally been used in gastronomy in various cultures, such as European, Asian, East Indian, Victorian English, and Middle Eastern [24]. Up to the present, EF have been greatly used as garnish in high-end foodservice establishments. However, their potential is considerably greater. EF can be used fresh as a garnish or as an integral part of a dish, for example in salads, but some other applications include stuffing or use in stir-fry dishes. Flowers can also be used to add color and flavor to foods, for example soups, entrées, desserts or drinks. Finally, EF have also been introduced into processed foods to add diversity and innovation to the food market, besides the nutritional and health benefits for consumers [2,24,25]. 

This led us to investigate the knowledge and habits relating to EF in Portugal and Costa Rica, which have different social and cultural backgrounds. Table 3 shows the results obtained for the whole sample, separated by country, as well as the results of the chi-square tests made to evaluate if there were statistically significant differences between countries in relation to various aspects of information linked with EF. The first aspect investigated was whether the participants knew what EF were. The results of this survey showed that the great majority of the participants had already heard about EF (86.9%), particularly in Portugal where almost everyone knew about EF (96.7%), while in Costa Rica that percentage was lower, but still high (76.3%). The differences between both countries were statistically significant (*p* < 0.0005) and there was a moderate association between these two variables, country and having heard about EF (V = 0.302). 

A good level of information is fundamental to help make food decisions in general, and this is even more true when it comes to EF. Hence, some of the aspects investigated aimed to evaluate the degree of knowledge that the participants had about different aspects related with EF. In appearance, EF are similar to ornamental flowers, since many of the species are used in both contexts. They are beautiful but also interesting from the organoleptic and nutritional point of view while at the same time being safe for human consumption. Nevertheless, if it is true that all edible species can be used for decorative purposes, the contrary is not true, and it is crucial to distinguish them for their edibility by using chemical and biological parameters [1,26]. The results obtained showed differences between Portugal and Costa Rica in relation to some aspects that measure the level of knowledge about EF. This has been reported previously by Rodrigues et al. in [25]. Despite the attention that EF have been gaining in the past years, it appears that they are not popular for consumption in Latin America, being considered unfamiliar to some cultures in the American continent. Information and knowledge about EF are fundamental to help consumers understand whether they can be used and how, as well as what benefits they provide from the organoleptic, nutritional or even medicinal points of view [4,27,28].

As for the question regarding the availability of information about EF, very similar results were found in both countries, with practically all participants stating that they considered the information about EF as not enough (about 96%) (Table 3). For this question, no significant differences were found between countries. 

Aspects linked to safety and possible risks involved in the consumption of EF were also investigated. The obtained results indicated that only about one third of the participants (35.1%) considered that there were risks associated with the consumption of EF; specifically, the Portuguese participants were more aware of this possibility (42.4%) than Costa Rican participants (27.4%) (Table 3). Furthermore, a very important percentage of participants (33.8%) could not express an opinion about this fact, replying maybe or I do not know. The differences found between countries were statistically significant (*p* = 0.025) but the association was weak (V = 0.159). 

In the 21st century, the agro-food industry faces many challenges, including food security (the need to provide enough food to be consumed) and food safety (products that are safe to eat). EF meet these challenges and additionally they allow for introducing innovative products with nutraceutical properties and health benefits to the market [26,29]. Regarding the food safety domain, EF pose some additional concerns. While purely decorative flowers sometimes have toxic components that can lead to intoxication or even death, the flowers used for human consumption have to be absolutely safe. Additionally, in many cases, the cultivation of decorative flowers involves the use of harmful chemicals, whereas EF, aimed for gastronomic purposes or other consumption options, such as medicinal preparations, for example, are usually obtained through organic production [6]. Seeing as two main aspects linked to the safety of EF are important, specific questions were formulated to evaluate if the participants considered toxicity and/or pesticides as risks associated with EF consumption. In fact, regarding these questions the great majority of participants assumed they believed toxicity (83.8%) and pesticides (80.9%) to be effective risks when consuming EF (Table 3). Again, very marked differences were encountered between participants from Portugal and Costa Rica, with the percentages for those who consider these as risks being much higher in the Portuguese sample (93.5% and 91.8%, respectively, for toxicity and pesticides) as compared with Costa Rica (71.2% and 68.3%, respectively). These differences were significant (*p* = 0.001 and *p* < 0.0005, respectively, for Portugal and Costa Rica) and the associations between variables were moderate in both cases (V = 0.300 and V = 0.298, respectively). One last question investigated if the participants considered that other risks could be associated with EF, and in this case the percentage of agreement was lower. Here, 50.5% of participants believed other risks could be involved in the consumption of EF, again, the higher percentage being Portugal, with significant differences between both countries (*p* = 0.035) but a relatively weak association (V = 0.190).

As well as hypothesizing that some professionals working in some specific areas could possibly have a different pattern of answers when compared with the common participants, it was also investigated if there were differences between participants not related and those related with the following areas: Agriculture, Food and Nutrition, Hotels and Restaurants. These results are presented in Table 4 where Portugal and Costa Rica are considered together.

The results in Table 4 show significant differences (*p* < 0.0005) between the participants related within the professional areas previously specified and those who were not related for the question about having already heard about EF, with a significantly higher percentage of yes for the participants related (94.7% against 22.2%, respectively, for related and not related). Furthermore, the association between being or not being related with the specified professional areas and having heard about EF was moderate (V = 0.250). Regarding the question of whether the participants considered if there is enough information about EF, no significant differences were found, and in both groups the percentage of participants that considered the information to be scarce was very high. 

In the question regarding the risk associations of EF, participants with professional links with Food and Nutrition, Agriculture or Hotels and Restaurants are more aware of the risks in general (46.7% against 22.2% for those not related), more alert to the risk of toxicity (86.7% against 77.8%) but not to the risk of pesticides (80.5% against 81.3%) or other risks (50% for both). Nevertheless, these differences were not statistically significant. 

EF bring interesting elements to culinary and dietary habits; therefore, chefs find in EF valuable allies to their gastronomic preparations due to their aromas and bouquet, their color, shapes and the sensations they evoke [30]. Because of their nutritive and bioactive compounds, such as polyphenols and their antioxidant properties, they also contain important dietary elements with nutritional and health benefits, recognized by the professionals linked with nutrition and health [31,32]. The cultivation of EF has to obey special requirements, usually in organic mode, and farmers are expected to be aware of the effects of the utilization of chemical products in their farms [6,30]. Nevertheless, despite these aspects, it was found that the professional area did not significantly impact the level of knowledge about EF.

### 3.2. Use of Edible Flowers

**Figure 1:**
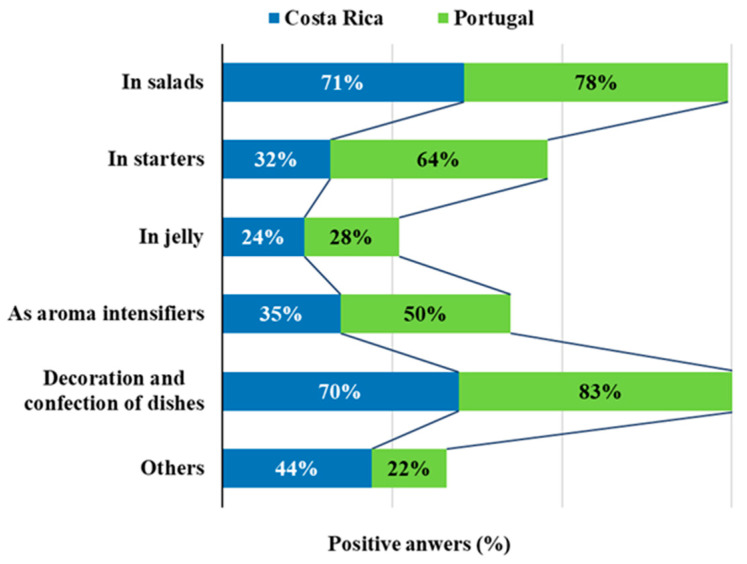
Possible ways in which the participants consumed edible flowers.

About half of the participants in this survey (47.6%) had already consumed EF, and this percentage was very similar for both countries (47.7% and 47.5% for Costa Rica and Portugal, respectively). For those who had already consumed EF, it was asked in what ways and/or for what purposes they consumed EF. The results showed that 77% had consumed EF for decoration and confection of dishes, 75% had consumed EF in salads, 49% in starters, 43% as aroma intensifiers, 26% in jellies and 33% in other non-specified possibilities. Figure 1 shows the ways consumption differentiated by country and similar results were found for the use of EF as decoration and confection of dishes (70% for Costa Rica and 83% for Portugal) as well as in salads (71% for Costa Rica and 78% for Portugal), these being the most frequent forms of consumption in both countries. The use in jellies showed the lowest incidence in both countries (less than 30%) but the use in starters and as aroma intensifiers was more pronounced among Portuguese participants (64% and 50%, respectively) than among those from Costa Rica (32% and 35%, respectively). Although traditionally EF have been mainly used due to their smell and visual appeal, new and innovative value has been attributed to flowers as part of the food market and chefs’ resources. Apart from being consumed fresh or cooked, they can be used in savory dishes containing meat and fish, in soups and drinks (such as wine, beer, vinegar or spirits), in desserts, sweets, jellies, as well as spices, and dyes. Furthermore, they can also be used in a dried form, ground into powder, crystallized or used as foams in molecular gastronomy [2,4].

**Figure 2:**
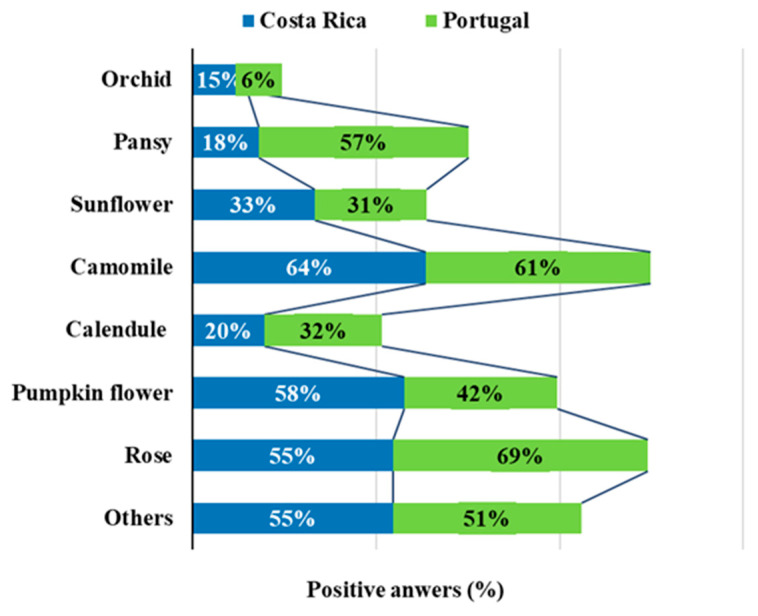
Types of flowers consumed by the participants.

One other question of the survey investigated which flowers the participants had already consumed. There are many flowers that are safe for human consumption and their usage can be varied according to availability and the chef’s discretion. Some of the most frequently reported EF include violet, pansy, marigold, rose, daisy, chicory, gardenia, hibiscus or jasmine, to name a few [6,32], and those most consumed by the participants in this study are included in this list. In this study, the most consumed flowers were chamomile and rose (by 62% of the participants in both cases). Then came the pumpkin flower (49%), pansy (38%), sunflower (32%), calendula (26%), orchid (10%), and others not specified were consumed by 53% of the participants. The results shown in Figure 2, which separates the consumption by country, indicate that most of the flowers which are popular in Costa Rica are also popular in Portugal and vice-versa: chamomile (64% and 61%, respectively, for Costa Rica and Portugal) and rose (55% and 69%, respectively) as the most popular, and orchid (15% and 6%, respectively) as the least. The usage of EF is very much dependent on tradition on one side, but also on innovation and new gastronomic trends on the other. While in Europe and Asia there is an old tradition linked with the usage of flowers for human consumption which is well documented, in some other parts of the world this habit is not so present among the population. Additionally, European chefs are among those who use creativity to explore the application of EF into their recipes [5,25], and this helps to explain some differences found when comparing the usage of EF in Portugal and in Costa Rica.

Regarding the frequency of consumption, 94% consumed EF sporadically (100% of the participants from Costa Rica and 88% of the participants from Portugal) and only 6% consumed them regularly. Some differences could be observed between the two countries, while in Portugal 12% already consume EF on a regular basis, in Costa Rica no one does. 

**Figure 3:**
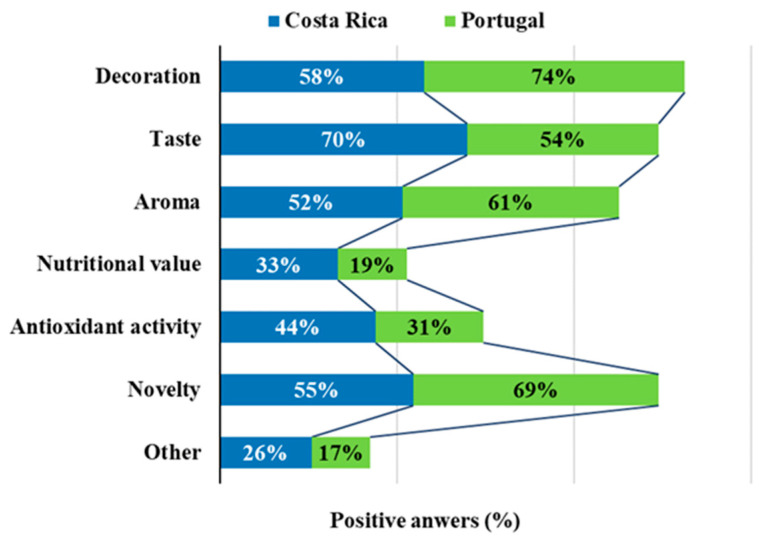
Reasons pointed out by the participants for consumption of EF.

Decoration, novelty, taste and aroma were cited as the most relevant reasons to use EF, while nutrition and antioxidant activity have the lowest importance. Nevertheless, the nutritional importance of many different EF has been established, as well as the value associated with some of the bioactive compounds that they contain that bear biological activities beneficial for human health—for example, the high antioxidant activity provided by phenolic compounds [6,33,34]. The reasons why participants consumed EF were varied and included their ability to decorate dishes or other food preparations (66%), their exquisite taste (62%), the perception of novelty associated with their utilization (62%), their aroma and bouquet (57%), their bioactive compounds that bear antioxidant activity (37%), their nutritional value, most especially regarding micronutrients lime vitamins and minerals (26%), among others (21%). Figure 3 represents the reasons indicated by the participants, separated by country, and some differences could be identified. While Portuguese consumers value more decoration (74%) and novelty (69%) those from Costa Rica value more taste (70%) followed by decoration (58%). In addition, differences were found for the value of nutrition and antioxidant properties, which were much more prized by the consumers in Costa Rica (33% and 44%, respectively) than in Portugal (19% and 31%, respectively). 

**Figure 4:**
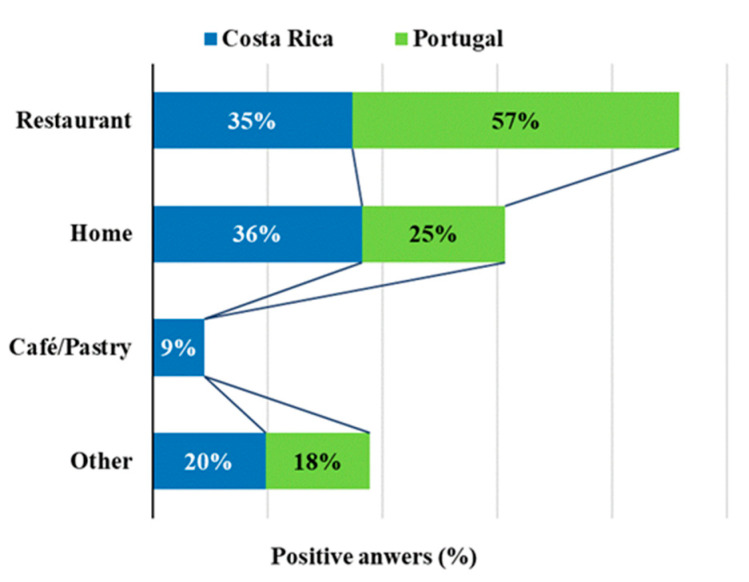
Places where participants consumed EF for the first time.

The places where the participants ate EF for the first time were in restaurants (for 46%), followed by the participants’ own homes (30%) and only a small percentage ate them for the first time in cafés or pastry shops (4%). According to Figure 4, the first consumption for the Portuguese occurred mostly at restaurants (57%) and with a lower expression at home (25%), while in Costa Rica both places were even (35% and 36%, respectively, for homes and restaurants). As chefs know already that some aspects linked with culinary pleasure captured through our senses are closely connected to emotions, they explore this by using ingredients and raw materials which are able to awaken these sensations. Hence, flowers, due to their varied colors, intense aromas and exquisite taste, are among those ingredients, and restaurants are, therefore, places where EF can be found in gastronomic preparations [35,36].

**Figure 5:**
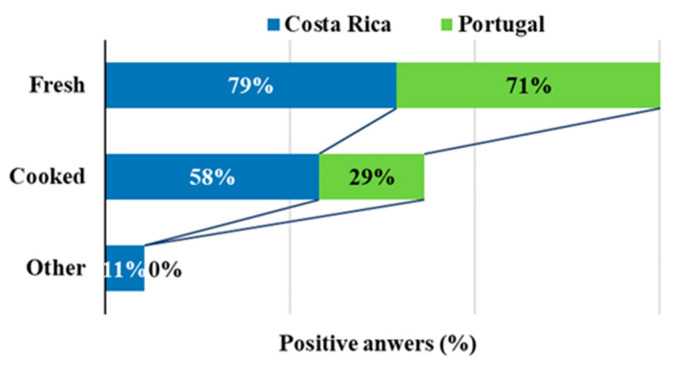
Forms of consumption of EF.

It was observed that 29% of the participants used EF for gastronomic preparations, this percentage being equal in both countries. Additionally, it was observed that the fresh state was the preferred form of consumption, by 75% of participants, while 43% used them cooked. Some differences were found between countries for the utilization of EF in the cooked form, being more expressive among consumers in Costa Rica (58%) than in Portugal (29%) (Figure 5). 

The marketing of fresh EF poses some challenges as both dehydration and oxidation make them highly perishable, diminishing their nutritional and bioactive characteristics. On the other hand, they are easily contaminated by insects, thus compromising their safety while also decreasing their attractiveness. For these reasons, fresh edible flowers are sold packed in rigid plastic containers [4]. Regarding the places where consumers obtain EF, most participants buy them in supermarkets (43%) or collect them in the wild (58%) or grow them at home (53%). Still a worrying fraction admit buying EF in flower shops (13%), which is highly critical, since ornamental flowers may not, and many times do not, comply with the necessary quality criteria to be ingested as foods [1]. Flowers can carry traces of pesticides and insects, and therefore, flowers grown for ornamental purposes may be among the edible species but should not be used for human consumption because they may be contaminated with pesticides or special fertilizers for better blooming. The flowers used in gastronomy should be cultivated purposely by the cook who will use them or bought from organic vegetable producers, as safe foods [5].

**Figure 6:**
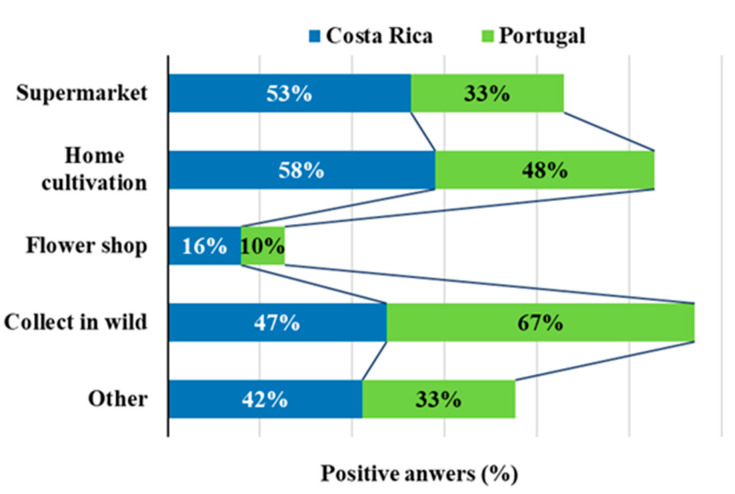
Places where the participants buy EF.

Some differences were found also for this question among participants from different countries (Figure 6), since in Costa Rica home cultivation is the most frequent way to obtain EF (58%), followed by the supermarket (53%) and then collection in the wild (47%), while in Portugal the most expressive way to obtain EF is to collect them from the wild (67%), then home cultivation (48%) and the supermarket comes in third (33%). The buying of EF in flower shops is more pronounced in Costa Rica (16%) compared to Portugal (10%).

**Figure 7:**
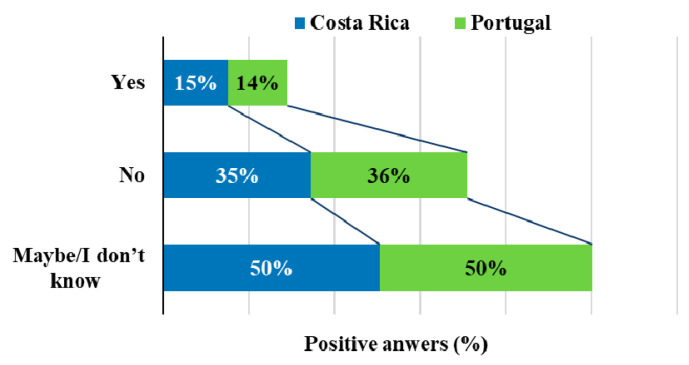
Availability of EF.

Another aspect investigated in this study, related to the ease of obtaining EF, is only 15% consider it is easy to obtain EF, while 36% think not, but almost half of the participants (49%) think maybe or do not know. These results were very similar for both countries, as indicated in Figure 7. As EF are still a niche in the food market, it is natural that their availability is lower when compared with other food categories such as fruits and vegetables [5].

**Figure 8:**
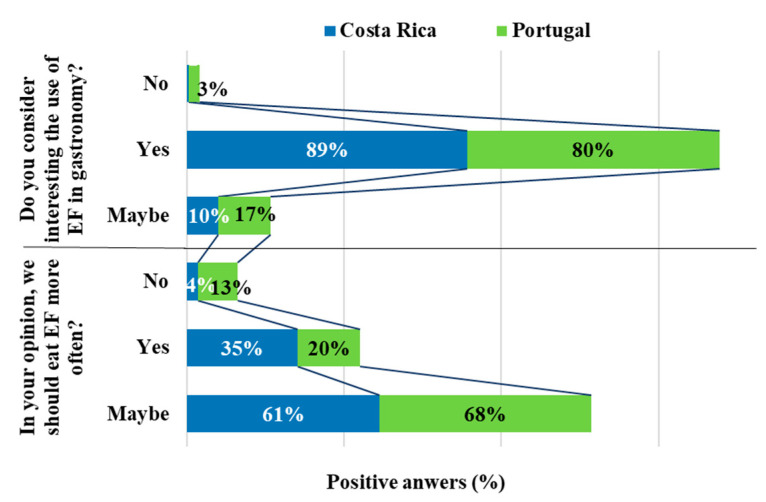
Opinions about the use of EF for culinary purposes.

Finally, two questions were dedicated to gathering the participants’ opinions about the usage of EF in gastronomy. The results obtained showed that most participants (85%) consider the use of EF for gastronomic purposes interesting, particularly those from Costa Rica (89%) with a higher expression than Portugal (80%) (Figure 8). Regarding the second question, only 27% believe that we should eat EF more often, being a higher percentage for participants from Costa Rica (35%) than Portugal (20%).

### 3.3. Effect of Sociodemographic Variables in Edible Flowers’ Consumption

DFA is used to determine which variables discriminate between two or more naturally occurring groups. In this study, DFA was used to determine which variable(s) are the best predictors for the consumers’ subsequent choice. The discriminant function is a linear function of the predictor variables such that it can discriminate between groups of respondents, i.e., differentiate low probability or high probability for a specific consumption attitude group. This is estimated by including all the predictors simultaneously and using the direct method of discriminant analysis.

For the question “How did you eat the flowers?” it was observed that education level, living environment and age group do not influence the consumers’ attitudes (Table 5). Nevertheless, statistical differences were found between sex groups for the use of EF in decoration and confection of dishes (female participants eat the flowers more in decoration and confection of dishes than male participants do). The differences observed between the two countries were assigned by the different uses of EF in starters.

Concerning the question “Which flowers have you already eaten?” for both age group and education level, no significate differences were observed (Table 5). However, significant differences were found in the pansy and pumpkin flower consumption in both countries. Additionally, it was further observed that the pansy flowers were consumed differently according to gender. It was observed also that the living environment influences the consumption of pumpkin flowers and roses.

For the frequency of the consumption of EF, only in the country and education level groups were significant differences found. For almost all sociodemographic groups studied, the motives for the consumption of EF did not present statistical differences. Conversely, the age group presented statistical differences concerning the use as decoration and for nutritional purposes (Table 5). The way the participants in each country consume flowers is significantly different when it comes to the consumption in the fresh form or cooked. This result is in accordance with the aforementioned statistic discussed in Figure 5, which shows that participants from Costa Rica eat cooked EF in much higher percentages than those from Portugal.

For the question “If you use EF, where do you buy them?” significant differences were found only for the living environment variable and regarding the option “others”, which accounts for other ways to buy EF not specified in the answering possibilities (Table 5). Furthermore, no significant differences were found in the question “Is there enough information about EF?” for country, age group, sex and education level variables. In this case only the different sex groups showed different opinions about the availability of information on EF. Regarding the risks associated with the consumption of EF, only for the risks associated with pesticides were there differences found in the participants from both countries and for both sexes (Table 5).

For the questions “EF are easy to obtain?” and “In your opinion we should eat EF more often?” no significant differences were found for all sociodemographic groups.

## 4. Conclusions

The present work concluded that the level of information about EF is significantly different between participants form the two countries evaluated, namely Costa Rica and Portugal. The highest differences were found in the knowledge about EF, or the risks associated with their consumption, particularly concerning toxicity and pesticides, for which the associations were moderate. As for the differences in information considering different professional groups, no significant differences were found except for having heard about EF, for which the association was moderate.

Regarding the use of EF, they are used mainly for decoration and confection of dishes and in salads, and those most consumed are rose and chamomile, which are consumed sporadically. Reasons for the consumption of EF include decoration, novelty, taste and aroma. Most participants ate EF for the first time in restaurants, and they consume them mostly fresh. In regard to obtaining EF, it consists mainly in collecting them in the wild, followed by home cultivation and then supermarkets, but consumers believe it is quite difficult to acquire. A great majority of the participants believe that the use of EF for gastronomic purposes is interesting, but a much smaller fraction believe that we should eat EF more often.

Finally, DFA revealed that country was the variable for which the differences in the consumption of EF between groups was more pronounced, while education level and age group showed the lowest variability between groups.

As our sample was recruited by convenience, more women and more people with university degrees volunteered to participate in the survey, and this constitutes one limitation of the study, also considering the limited number of responses obtained. However, this study is a first approach to understanding the habits of EF consumption in these two countries, highlighting which aspects are more relevant for the consumption of this type of product, from the point of view of the consumer. Additionally, this study allows for some complementing future lines of work, namely the importance of safety assurance, quality control and marketing issues, including labelling as a way to protect and inform consumers.

## Figures and Tables

**Table 1:** Sociodemographic characterization of the study sample.

Variable		Costa Rica	Portugal	Total ^(1)^
Dimension: N (%)		139 (47.9)	151 (52.1)	290 (100)
Age ^(2)^ (MV ± SD years)		38 ± 12	41 ± 13	40 ± 13
Age group	Young adults (18–30 years) (%)	30.2	23.8	26.9
	Middle aged adults (31–50 years) (%)	52.6	53.0	52.7
	Senior adults (51–65 years) (%)	15.8	20.5	18.3
	Elderly (≥66 years) (%)	1.4	2.6	2.1
Sex	Women (%)	62.6	80.8	72.1
	Men (%)	37.4	19.2	27.9
Education level	Basic (%)	2.9	3.3	3.1
	Secondary (%)	12.2	15.2	13.8
	University (%)	84.9	81.5	83.1
Living environment	Urban (%)	66.2	68.0	67.2
	Suburban (%)	12.9	7.3	10.0
	Rural (%)	20.9	24.7	22.8

^(1)^ Combined results of Portugal and Costa Rica. ^(2)^ Age expressed as mean value (MV) ± standard deviation (SD).

**Table 2:** Professional area of the participants.

Professional Area	Costa Rica		Portugal		Total ^(1)^	
	Yes (%)	No (%)	Yes (%)	No (%)	Yes (%)	No (%)
Nutrition/Food	17.3	82.7	58.0	42.0	38.4	61.6
Agriculture	31.2	68.8	29.3	70.7	30.2	69.8
Hotels/Restaurants	5.0	95.0	12.1	87.9	8.7	91.3
Not related to any of the above	43.5	56.5	38.3	61.7	47.0	53.0

^(1)^ Combined results of Portugal and Costa Rica.

**Table 3:** Information about edible flowers (EF), according to country.

Question		Total ^(1)^	Costa Rica	Portugal	CST ^(2)^		CC ^(3)^
					χ^2^	*p*	V
Have you heard about EF?	Yes (%)	86.9	76.3	96.7	26.530	<0.0005 ^(5)^	0.302
	No (%)	13.1	23.7	3.3			
Do you think there is enough information about EF?	Yes (%)	3.8	3.6	4.0	0.028	0.557 ^(5)^	-
	No (%)	96.2	96.4	96.0			
Do you think there are risks associated with consumption of EF?	Yes (%)	35.2	27.4	42.4	7.342	0.025	0.159
	No (%)	31.0	33.8	28.5			
	M ^(4)^ (%)	33.8	38.8	29.1			
Do you think toxicity is a risk?	Yes (%)	83.8	71.2	93.5	12.237	0.001 ^(5)^	0.300
	No (%)	16.2	28.8	6.5			
Do you think pesticides are a risk?	Yes (%)	80.9	68.3	91.8	12.105	<0.0005 ^(5)^	0.298
	No (%)	19.1	31.7	8.2			
Do you think there are other risks?	Yes (%)	50.5	39.6	58.7	3.995	0.035	0.190
	No (%)	49.5	60.4	41.3			

^(1)^ Combined results of Portugal and Costa Rica. ^(2)^ CST: chi-square test (level of significance of 5%: *p* < 0.05) for country differences. ^(3)^ CC: Cramer’s coefficient, only indicated if there were significant differences. ^(4)^ This option accounts for Maybe/I do not know. ^(5)^ Fisher’s exact test.

**Table 4:** Information about edible flowers (EF), according to professional areas in Portugal and Costa Rica (considered together).

Question		Related ^(1)^	Not Related ^(1)^	CST ^(2)^		CC ^(3)^
				χ^2^	*p*	V
Have you heard about EF?	Yes (%)	94.7	22.2	17.901	<0.0005 ^(5)^	0.250
	No (%)	5.3	77.8			
Do you think there is enough information about EF?	Yes (%)	5.3	2.2	1.794	0.151 ^(5)^	-
	No (%)	94.7	97.8			
Do you think there are risks associated with consumption of EF?	Yes (%)	46.7	22.2	19.234	0.442	-
	No (%)	27.0	35.6			
	M ^(4)^ (%)	26.3	42.2			
Do you think toxicity is a risk?	Yes (%)	86.7	77.8	1.738	0.142 ^(5)^	-
	No (%)	16.3	22.2			
Do you think pesticides are a risk?	Yes (%)	80.5	81.3	0.012	0.552 ^(5)^	-
	No (%)	19.5	18.7			
Do you think there are other risks?	Yes (%)	50.0	50.0	0.000	0.579 ^(5)^	-
	No (%)	50.0	50.0			

^(1)^ Related or not related with the following areas: Nutrition/Food, Agriculture, Hotels/Restaurants. ^(2)^ CST: chi-square test (level of significance of 5%: *p* < 0.05) for country differences. ^(3)^ CC: Cramer’s coefficient, only indicated if there were significant differences. ^(4)^ This option accounts for Maybe/I do not know. ^(5)^ Fisher’s exact test.

**Table 5:** Discriminant function analysis summary (*p*-value).

Questions		Country	Age Group	Sex	Living Environment	Education Level
How did you eat the flowers?	In salads	ns	ns	ns	ns	ns
	In starters	0.0019 **	ns	ns	ns	ns
	In jelly	ns	ns	ns	ns	ns
	As aroma intensifiers	ns	ns	ns	ns	ns
	Decoration and confection of dishes	ns	ns	0.0307 *	ns	ns
	Others	0.0232 *	ns	ns	ns	ns
Which flowers have you already eaten?	Orchid	ns	ns	ns	ns	ns
	Pansy	0.0001 ***	ns	0.0125 *	ns	ns
	Sunflower	ns	ns	ns	ns	ns
	Chamomile	ns	ns	ns	ns	ns
	Calendula	ns	ns	ns	ns	ns
	Pumpkin flower	0.0405 *	ns	ns	0.0007 ***	ns
	Rose	ns	ns	ns	0.0209 *	ns
	Others	ns	ns	ns	ns	ns
Frequency of consumption of EF?		0.0021 **	ns	ns	ns	0.0199 *
What motivates you to consume them?	Decoration	ns	0.0287 *	ns	ns	ns
	Taste	ns	ns	ns	ns	ns
	Aroma	ns	ns	ns	ns	ns
	Nutrition	ns	0.0346 *	ns	ns	ns
	Antioxidant activity	ns	ns	ns	ns	ns
	Novelty	ns	ns	ns	ns	ns
	Other	ns	ns	ns	ns	ns
If you consume EF, in what form?	Fresh	0.0251 *	Δ	ns	ns	Δ
	Cooked	0.0053 **		ns	ns	
	Other	ns		ns	ns	
If you use EF, where do you buy them?	Supermarket	ns	Δ	ns	ns	Δ
	Home cultivation	ns		ns	ns	
	Flower shop	ns		ns	ns	
	Collect in wild	ns		ns	ns	
	Other	ns		ns	0.0051 **	
Is there enough information about EF?		ns	ns	0.0396 *	ns	ns
Risks associated with consumption of EF	Toxicity	ns	ns	ns	ns	Δ
	Pesticides	0.0055 **	ns	0.0032 **	ns	
	Other	ns	ns	ns	ns	
EF are easy to obtain?		ns	ns	ns	ns	ns
In your opinion we should eat EF more often?		ns	ns	ns	ns	ns

EF: edible flowers. ns: *p* > 0.05; * 0.01 < *p* < 0.05; ** 0.001 < *p* < 0.01; *** *p* < 0.001. Δ—some groups contain only a valid case.
